# Effects of Cognitive Behavioral Therapy on Eating Behaviors, Affective Symptoms, and Weight Loss After Bariatric Surgery: a Randomized Clinical Trial

**DOI:** 10.1007/s11695-018-3471-x

**Published:** 2018-08-15

**Authors:** Jøran Hjelmesæth, Jan H. Rosenvinge, Hege Gade, Oddgeir Friborg

**Affiliations:** 1 Morbid Obesity Centre, Department of Medicine, Vestfold Hospital Trust, Boks 2168, 3103 Tønsberg, Norway; 2 Department of Endocrinology, Morbid Obesity and Preventive Medicine, Institute of Clinical Medicine, University of Oslo, Oslo, Norway; 3 Faculty of Health Sciences, Department of Psychology, UiT – The Arctic University of Norway, Tromsø, Norway

**Keywords:** Cognitive behavioral therapy, Weight loss, Eating behaviors, Depression, Health-related quality of life

## Abstract

**Background:**

The long-term effects of presurgical psychological interventions on weight loss, eating behaviors, affective symptoms, and health-related quality of life remain uncertain. This study aimed to assess the 4-year effects of cognitive behavioral therapy (CBT) before bariatric surgery on these outcomes.

**Methods:**

Single-center randomized controlled parallel-group trial. Patients were assessed after CBT before bariatric surgery (*n* = 98) and 1 year (*n* = 80) and 4 years (*n* = 61) after surgery. The intervention group received a 10-week preoperative individual CBT focusing on self-monitoring to identify triggers of dysfunctional eating behaviors in order to improve regulation of eating as well as the breaking of the interrelationship between eating behaviors, negative mood, and dysfunctional cognitions.

**Results:**

The 61 patients (70% women) had a mean (SD) age of 42.4 (10.1) years and BMI 43.5 (4.4) kg/m^2^. Preoperative CBT was not associated with 1- and 4-year reduction of dysfunctional eating behaviors, affective symptoms and body weight, or improved health-related quality of life. Patients with minor or considerable symptoms of depression receiving CBT had lower mean BMI than controls, both before surgery, − 1.1 kg/m^2^, and − 1.5 kg/m^2^, and 4-years after surgery, − 2.9 kg/m^2^ and − 7.5 kg/m^2^, respectively.

**Conclusion:**

Presurgical CBT was not associated with better long-term outcomes. However, in patients with minor or considerable symptoms of depression, CBT was associated with lower body weight before and 4 years after surgery. Additional studies are required to verify whether patients with symptoms of depression should be offered CBT before and/or after bariatric surgery, and which clinical aspects the CBT should address.

**Trial Registration:**

Clinicaltrials.gov Identifier: NCT01403558.

## Introduction

Mental health and eating behaviors may improve after bariatric surgery, and these beneficial effects may affect weight loss and maintenance [1–6]. However, these benefits do not seem to endure beyond the years immediately after surgery [7, 8], and bariatric surgery has been associated with increased long-term risk of new onset of depression, anxiety, and sleep disorders [9]. Furthermore, although the average long-term weight loss after bariatric surgery is satisfactory, the general reporting of mean weight values probably underestimates the extent of weight regain [10]. Importantly, emotional and uncontrolled eating, as well as anxiety or depression, may increase weight regain due to an association between these mental health symptoms and poor weight loss [6, 11].

Preoperative medical weight management may promote postoperative outcomes, but the empirical evidence is mixed [12]. More specifically, few clinical studies have evaluated the short- to medium-term effects of presurgical behavioral interventions on postsurgical weight loss [13–15], while the long-term (≥ 2 years) effects of presurgical psychological interventions on weight loss and mental health outcomes remain uncertain [16]. We previously found, however, in a randomized controlled trial (RCT) that a 10-week preoperative cognitive behavioral therapy (CBT) significantly reduced dysfunctional eating behaviors, affective symptoms, and body weight before surgery [17], but that these effects disappeared at 1-year follow-up [18]. A smaller pilot RCT demonstrated that six sessions of telephone-based CBT before bariatric surgery was associated with improved eating psychopathology and depression [19]. In addition, a retrospective chart review study showed that a brief cognitive behavioral group psychotherapy was associated with reduced binge eating behaviors among bariatric surgery candidates [20]. Although it has been hypothesized that postoperative eating adaptation and depression may affect later weight loss, the evidence is insufficient [21].

The present 4-year follow-up of a previously published randomized controlled study [17] aimed to assess whether a 10-week presurgical CBT intervention implied favorable long-term improvements in eating behaviors, weight loss, affective symptoms (anxiety and depression), and health-related quality of life (HRQoL). In addition, it explored whether changes in the mental health status (i.e., affective symptoms, notably depression), as well as mental health-related vulnerability (neuroticism) and protective factors (resilience), modified the treatment response in terms of body weight (BMI) in the follow-up period. We hypothesized that a 10-week presurgical CBT intervention would improve long-term eating behaviors, weight loss, and affective symptoms.

## Materials and Methods

### Trial Design, Setting, and Participants

This 4-year follow-up of a single-center parallel-group RCT (http://clinicaltrials.gov/ct2/show/NCT01403558) was conducted at a tertiary care center in Norway [17, 18]. Patients preparing for bariatric surgery were enrolled from September 2011 to June 2012. Measurements were carried out 4 months and 4 weeks before bariatric surgery (T0 and T1), and at 1 and 4 years (T2 and T3) after surgery. All participants signed an informed consent and were randomly assigned to CBT or standard preoperative care in a 1:1 ratio [17].

### Interventions

The interventions have been described in detail [17]. Briefly, all patients were offered voluntary consultations from either a medical doctor, a dietician, a nurse, or a physical therapist tailored to the patients’ individual needs. In the psychological treatment arm, each patient received an additional individual 10-week CBT intervention. This intervention focused on self-monitoring to identify triggers of dysfunctional eating behaviors in order to improve regulation of eating as well as the breaking of the interrelationship between eating behaviors, negative mood, and dysfunctional cognitions (Table 1). A total of 48 out of 50 patients randomized to intervention completed all sessions.

**Table 1:** Overview of the 10-week CBT intervention

Sessions	Session content
Session 1 (face-to-face) Both groups	• Establishing rapport with the patient in order to facilitate a good therapeutic working alliance. • Providing information about the interventions to all patients. • Conducting the baseline measurements and perform the randomization and informing the patients about their allocated group.
Session 2 (face-to-face)	• Introduction to the underlying principles of the therapy (working transparently, collaboratively, time-limited, and using a manual). • Informing the patient about CBT and the treatment plans in the study. • Psychoeducation focusing on the relationships between eating behaviors, cognitive and behavioral patterns, affect regulation, and obesity, thus introducing the patients to the CBT model. • Introducing and explaining homework sheets for sessions 3 and 4.
Sessions 3 + 4 (telephone)	• Reviewing the patient’s homework sheets. • Recognizing and addressing dysfunctional eating behaviors. • Working with the patient’s behavioral eating patterns (what triggers eating), and the associated cognitions and emotions. • Providing the patients’ means to assess their own perception about recognizing improvement in dysfunctional cognitions and eating behaviors.
Session 5 (face-to-face)	• Coping with situational “triggers” that may lead to dysfunctional cognitive and eating behavioral patterns. • Working with the patient’s cognitive and behavioral eating patterns (“triggers,” cognition, emotion, and eating behavior). • Introducing and explaining homework sheets for sessions 6 and 7
Sessions 6 and 7 (telephone)	• Reviewing the patient’s homework sheets. • Continuing the intervention techniques. • Reinforcing positive changes in eating behaviors.
Session 8 (face-to-face)	• Continuation or refining intervention techniques (as session 5) by guiding the patient in avoiding situational “triggers” and making a plan for practicing new eating behaviors. • Introducing and explaining home-work sheets for sessions 9 and 10.
Sessions 9 and 10 (telephone)	• Reviewing the patient’s home-work sheets. • Continuation or refining intervention techniques.
Session 11 (face-to-face)	• Relapse prevention. • Ending of treatment and helping the patient to maintain positive changes.

### Prespecified Outcomes

The primary outcomes were dysfunctional eating behaviors: emotional eating (EE), uncontrolled eating (UE), and cognitive restraint (CR). The secondary outcomes were weight loss and affective symptoms (depression and anxiety), and exploratory outcomes were HRQoL.

### Measurements

The clinical data were collected through a web-based solution (https://no.surveymonkey.com/ and https://metreno.com/). Body weight, height, eating behaviors, and symptoms of anxiety and depressions were measured at all time points (T0–T3). Personality traits were measured at baseline (T0), and resilience and HRQoL were measured at T2 and T3.

#### Dysfunctional Eating Behaviors

The Three-Factor Eating Questionnaire (TFEQ R-21) has been validated for use in individuals with obesity [22, 23]. It consists of 21 items comprising the 3 subscales: EE (6 items; Cronbach’s *α* = .92), UE (9 items; *α* = .73), and CR (6 items, *α* = .84). They were transformed to a 0–100 scale to become comparable. Higher scores indicate higher levels of dysfunction.

#### Anxiety and Depression

The Hospital Anxiety and Depression Scale (HADS) includes 7 items assessing non-vegetative symptoms of depression (HADS-D subscale) and 7 items assessing non-vegetative symptoms of anxiety (HADS-A subscale) [24]. Items are scored from 0 to 3 with a total score range 0–21 for each subscale. The reliability and validity of the HADS are well supported in Norwegian patient samples, and a cut-off score ≥ 8 is used to indicate a tentative diagnosis of depression or anxiety [25, 26].

#### Neuroticism

The Norwegian version of the NEO Personality Inventory-Revised (NEO PI-R) was used [27]. The NEO PI-R is based on the five-factor model (FFM) of personality [28]. Five trait domains are measured by 240 items on a 5-point Likert scale, where higher scores indicate more of the domains. In the present study, the domain neuroticism (*N*) was used.

#### Resilience

Mental health promoting resilience resources were assessed at the 1- and 4-year follow-ups (T2, and T3, respectively), using the Resilience Scale for Adults (RSA) [29]. It is a reliable and valid scale for assessing protection against hopelessness and depression when exposed to life stressors or adversity [30]. Higher scores imply better protection. A global RSA score was used in the present study, supported by a principal component analysis extracting a single eigenvalue > 1 (*λ* = 3.71, *R*^2^ = 61.9%).

#### Weight-Related HRQoL

The short form of impact of weight on quality of life (IWQOL-lite) is a 31-item questionnaire which assesses the impact of weight on quality of life with acceptable validity and reliability in patients with obesity (test-rest reliability *r* = 0.74–0.91; *α* = .85). It contains the five domains, i.e., physical function, self-esteem, sexual life, public distress, and work [31, 32]. An overall score was used, as a principal component analysis extracted a single component with an eigenvalue > 1 (*λ* = 3.21, *R*^*2*^ = 64%). This measure was used at T2 and T3.

#### Body Weight and BMI

A digital scale (Soehnle Professional 2755, http://www.soehnle.de/), a bioelectrical impedance analyzer (Tanita BC-418), and a wall-mounted stadiometer (Seca 240, http://www.stadiometer.com/) were used to measure body weight and height, respectively, and BMI was calculated. All patients were examined in light clothing and without shoes.

### Sample Size

The original sample size calculation required a minimum of 80 patients to detect ≥ 15% reduction in dysfunctional (emotional and uncontrolled) eating scores among at least 30% of the patients in the intervention, compared with none in the control group (based on power = 90%, alpha = 5%). Expecting a 20% withdrawal rate, 102 patients were recruited [17].

### Randomization

A block randomization procedure (www.randomizer.org) was employed (blocks of 4) to ensure balance between the groups. The allocation ratio was 1:1 [17].

### Ethics

The study was approved by the Regional Committee for Medical and Health Research Ethics (REK Sør-øst, ID no. 2010/2071), and it satisfies the Declaration of Helsinki standard [33].

### Statistical Methods

The longitudinal treatment response was examined with the mixed regression module as the restricted maximum likelihood function estimates unbiased model parameters using all the available data; thus, tolerating attrition of participants better than conventional analysis of variance methods. The pretest score was used as a covariate to increase the statistical power for detecting changes across the three posttest measures [34]. Questionnaire data: The dependency in the fitted residuals were accounted for by specifying a compound symmetry (constant correlations) or an autoregressive (falling correlations) matrix, depending on whichever model fit best (lowest Bayesian information criteria, BIC). BMI data: An unstructured covariance matrix had to be used to account for the large differences in the fitted residual variances (T2 and T3 considerably larger than T1), as well as the residual correlations (T2 and T3 were strongly correlated, whereas T1–T2 and T1–T3 were not). The robust method (Huber-White estimator) was used to yield a consistent estimate of this covariance matrix. Degree of freedom was computed using Satterthwaite approximation. A log link function was used to completely normalize the fitted residuals. The fixed factors were as follows: *group* (0 = control, 1 = CBT) representing the overall treatment effect, *time* (three posttest measures) representing change in the follow-up period, and the *group* × *time* interaction representing a particular treatment response at specific time points only. Differences between the treatment arms were examined with planned comparison at each time point (least square difference tests). Type III *F*-tests and an alpha level of *P* < .05 were used. Standardized group mean differences were calculated as Hedge’s $g=\frac{{\mathrm{EM}}_1-{\mathrm{EM}}_2}{} \times J\ \mathrm{correction}$, using the adjusted means in the nominator and the observed pooled SD for the specific time point in the denominator, as recommended by Feingold [35]. Effect sizes were interpreted as negligible, small, medium, or large for *d*’s *<* .20, .20–.49, .50–.79, or ≥ .80, respectively [36]. The patient background variables (gender and age), the primary (EE, UE, and CR) and the secondary (HADS-A and HADS-D) outcome variables, and vulnerability (neuroticism) and protective factors (resilience) were examined in separate regression models as moderators of the treatment effect (*M* × group and *M* × *group × time*). Their main and interaction with time effects (*M* and *M* × *time*) were included for adjustment purposes. Repeatedly assessed moderators were treated as time-variant variables (e.g., depression), whereas those recorded once were treated as time-invariant (e.g., gender). A significant moderation effect indicates that change in the moderator was associated with the treatment response (*M* × group), and whether it was particularly pronounced at certain time points (*M* × *group* × *time*). *P* values less than .05 were considered statistically significant. All analyses were performed using SPSS version 24 (SPSS Inc).

## Results

### Participant Characteristics

**Fig. 1:**
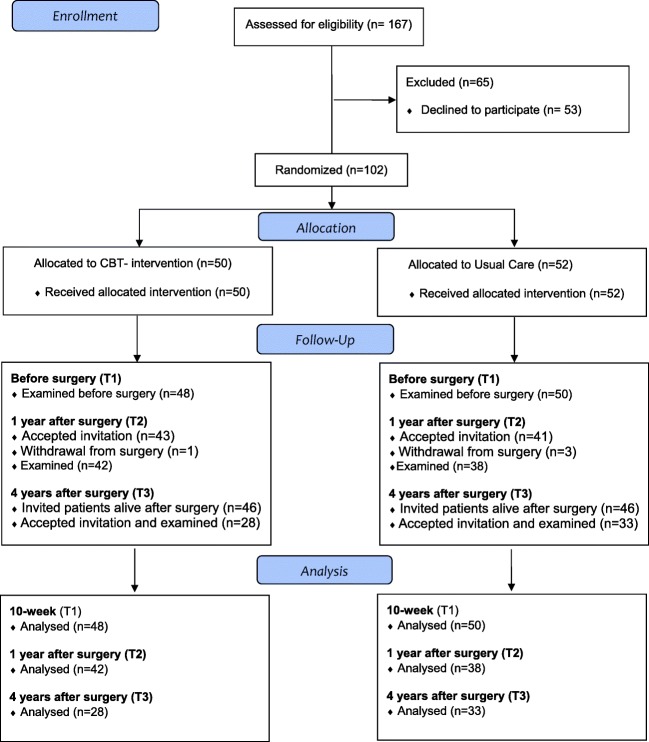
Participant flow through enrollment, allocation, and follow-up

As previously reported, 102 treatment-seeking white patients were allocated to either a 10-week presurgical CBT intervention or standard presurgical care (T0), and after exclusion of 2 patients in each treatment group due to trial fatigue, data from 98 patients were available for analysis before surgery (T1) (Fig. 1) [17]. After exclusion of 4 patients who did not undergo bariatric surgery and 2 who died, 92 patients were invited to participate in the present 4-year follow-up study, and 61 (66%) patients accepted the invitation. At baseline (T0), these 61 patients (70% women) had a mean (SD) age of 42.4 (10.1) years and a BMI of 43.5 (4.4) kg/m^2^ (Table 2). The patients who completed the 4-year follow-up did not differ significantly from those who did not, regarding gender distribution, age, BMI, dysfunctional eating, or affective symptoms.

**Table 2:** Baseline demographics of the 61 patients examined at 4-year follow-up

	Total (*n* = 61)	Intervention (*n* = 28)	Controls (*n* = 33)	*P* value
Female (yes)	43 (70%)	16 (57%)	27 (82%)	.05
Age (years)	42.4 (10.1)	44.4 (10.0)	40.6 (10.2)	.16
BMI	43.5 (4.4)	43.6 (4.5)	43.5 (4.4)	.97
Procedure				
Gastric bypass	49 (80%)	23 (82%)	26 (79%)	> .99
Sleeve	12 (20%)	5 (18%)	7 (21%)	

Data are presented as observed mean (SD) or number (%), independent samples test or Fisher’s exact test as appropriate

### Primary Outcomes

The three eating behaviors outcomes showed a comparable treatment response as all *group* effects (EE *F*_1,96_ = 4.82, *P* = .031; UE *F*_1,90_ = 11.01, *P* < .001; CR *F*_1,89_ = 12.77, *P* < .001) and *group × time* effects (EE *F*_2,154_ = 10.79, *P* < .001; UE *F*_2,152_ = 13.81, *P* < .001; CR *F*_2,146_ = 6.11, *P* = .003) were significant (Table 3). As previously reported, planned comparison showed a significant treatment response post-intervention (T1, all *P*’s < .001) with large statistical effect sizes. All these effects disappeared 1 year (T2) [18] and 4 years (T3) after surgery, as none planned comparisons were significant, and the effect sizes were small or negligible (Table 3). While the control group showed significantly higher emotional eating and uncontrolled eating scores than the CBT group at T1, the results were reversed at T3 showing that the control group had numerically lower (non-significant) mean scores than the CBT group.

**Table 3:** Estimated means for eating behaviors as primary outcomes, and BMI, anxiety, and depression as secondary outcomes

Outcome variables	Before surgery (T1, *n* = 98)			1 year after surgery (T2, *n* = 80)			4 years after surgery (T3, *n* = 61)		
	Mean (95% CI)	Mean difference (95% CI)	*g* (*P* value)	Mean (95% CI)	Mean difference (95% CI)	*g* (*P* value)	Mean (95% CI)	Mean difference (95% CI)	*g* (*P* value)
Emotional eating									
CBT	30.5 (25.1, 35.9)	− 18.5 (− 26.0, − 11.0)	.71 (< .001)	21.4 (15.6, 27.1)	− 7.3 (− 15.6, 1.0)	.31 (.09)	40.2 (33.3, 47.0)	5.8 (− 3.5, 15.1)	− .22 (.22)
Control	49.0 (43.8, 54.3)			28.6 (22.7, 34.6)			34.3 (28.0, 40.7)		
Uncontrolled eating									
CBT	30.4 (26.4, 34.3)	− 18.2 (− 23.8, − 12.7)	.97 (< .001)	17.8 (13.5, 22.0)	− 5.6 (− 11.7, 0.6)	.28 (.08)	30.0 (25.0, 35.2)	2.6 (− 4.4, 9.5)	− .16 (.47)
Control	48.6 (44.7, 52.5)			23.3 (18.9, 27.7)			27.5 (22.8, 32.2)		
Cognitive restraint									
CBT	68.9 (63.6, 74.2)	20.2 (12.8, 27.6)	.98 (< .001)	62.6 (56.9, 68.2)	5.9 (− 2.2, 14.0)	.30 (.16)	58.5 (51.8, 65.1)	6.1 (− 3.0, 15.2)	.38 (.19)
Control	48.7 (43.6, 53.9)			56.7 (50.9, 62.5)			52.4 (46.2, 58.6)		
BMI									
CBT	42.2 (41.8, 42.5)	− 1.36 (− 1.95, − 0.77)	.28 (< .001)	30.0 (28.9, 31.1)	0.47 (− 1.05, 2.00)	− .10 (.72)	32.0 (30.3, 33.8)	0.47 (− 1.05, 2.00)	− .05 (.76)
Control	43.6 (43.1, 44.1)			29.5 (28.5, 30.6)			31.7 (30.3, 33.2)		
Anxiety									
CBT	4.9 (4.1, 5.6)	− 2.4 (− 3.5, − 1.4)	.69 (< .001)	4.4 (3.6, 5.2)	− 1.8 (− 2.9, − 0.6)	.57 (.003)	6.8 (5.8, 7.7)	0.9 (− 0.4, 2.3)	− .24 (.16)
Control	7.3 (6.6, 8.1)			6.2 (5.3, 7.0)			5.8 (4.9, 6.7)		
Depression									
CBT	2.5 (1.6, 3.3)	− 2.6 (− 3.8, − 1.4)	.78 (< .001)	1.5 (0.9, 2.1)	− 0.5 (− 1.3, 0.4)	.24 (.26)	3.4 (2.4, 4.4)	1.6 (0.2, 2.9)	− .56 (.02)
Control	5.0 (4.2–5.9)			2.0 (1.4, 2.6)			1.9 (1.0, 2.8)		

Baseline means are as follows: emotional eating = 51.4, uncontrolled eating = 48.3, cognitive restraint = 45.2, BMI = 43.6 kg/m^2^, HADS-A = 6.7, HADS-D = 5.0. *g* means Hedge’s effect size, *P* value means probability value for the between-group difference test

### Secondary Outcomes

As previously reported, the CBT treatment reduced affective symptoms significantly and strongly before surgery (T1; Table 3). At 1-year follow-up (T2), the treatment benefit on depression disappeared, while the anxiety reduction benefit remained significant and of moderate statistical effect size (Table 3). At the 4-year follow-up (T3), all differences disappeared. Depression scores were even moderately higher in the CBT compared to the control arm despite the scores being low and within the normal range.

#### BMI

Neither *group* (*P* = .89) nor the *group × time* (*P* = .20) interaction was significant, whereas *time* was significant (*F*_2,67_ = 389.93, *P* < .001). Despite the non-significant interaction, planned comparisons showed a small but significant benefit of CBT on BMI at T1 as previously reported (Table 3) [17].

### Exploratory Outcomes

HRQoL did not differ significantly between groups, but the group × time effect was significant (*F*_1,59_ = 7.38, *P* = .009). Planned comparisons of the group means at 1-year follow-up (CBT = 89.4 and control = 86.9, mean difference = 2.5, 95% CI − 3.2 to 8.2, *P* = .39) and 4-year follow-up (83.3 and 88.3, mean difference = − 5.0, 95% CI − 11.2 to 1.2, *P* = .11) confirmed a zig-zag pattern.

### Moderator Analyses of BMI: Did Certain Subgroups Respond to the CBT Treatment?

**Fig. 2:**
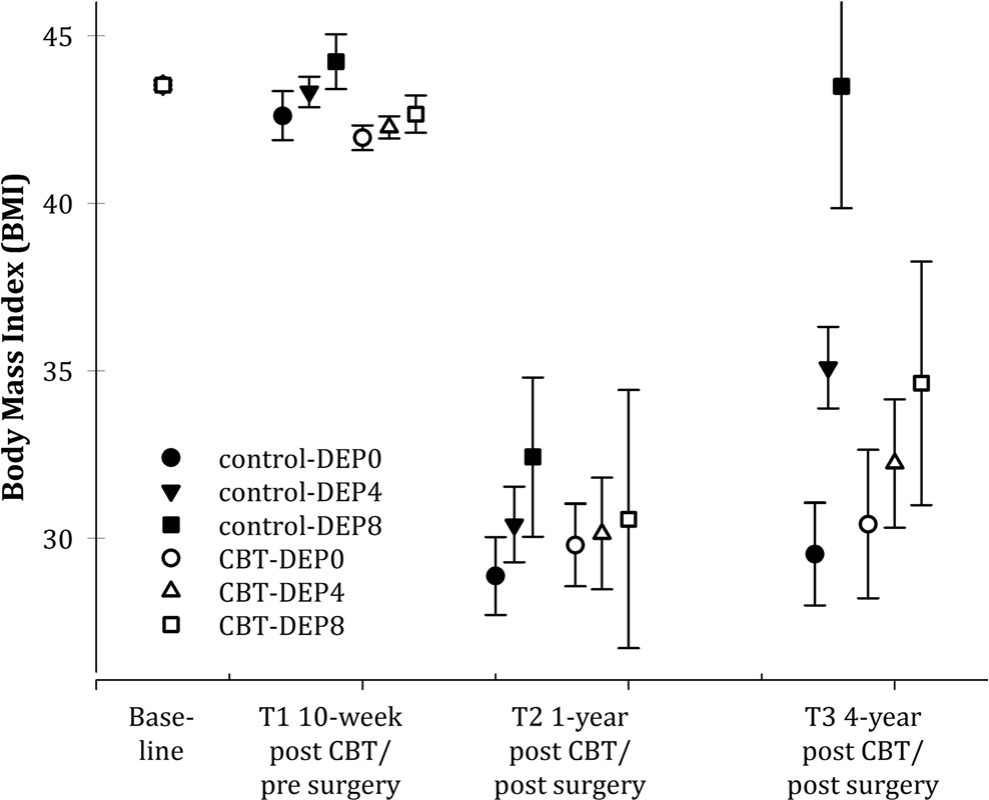
Estimated mean BMI (95% CI) after CBT and before surgery, and 1 and 4 years after surgery for patients with no symptoms of depression (DEP0), minor symptoms of depression (DEP4), and considerable symptoms of depression (DEP8)

Gender, age, eating patterns, HADS-A, and neuroticism did not significantly moderate the effect of CBT on BMI. Only resilience (RSA) and HADS-D emerged as significant moderators, of which RSA dropped out as non-significant if analyzed together with HADS-D. The final interaction model was thus the following: HADS-D (*F*_1,90_ = 29.82, *P* < .001), HADS-D × time (*F*_2,43_ = 12.30, *P < .001*), HADS-D × group (*F*_1,97_ = 7.87, *P* = .006), and HADS-D × group × time (*F*_2,43_ = 3.37, *P* = .044). Since HADS-D was continuously scored, planned comparison tests were based on estimated means for patients with absolutely no symptoms of depression (HADS-D = 0, standardized *Z*-score = − 0.92), minor symptoms of depression (HADS-D = 4, *Z* = 0.41), and considerable symptoms of depression (HADS-D = 8, *Z* = 2.06). Figure 2 shows that CBT was associated with a significantly lower BMI at T1 (before surgery) and T3 (4 years after surgery) among patients with HADS-D = 4 or HADS-D = 8; mean differences at T1 and T3 for those with HADS-D = 4, − 1.05 kg/m^2^ (95% CI, − 1.58 to − 0.52), and − 2.85 kg/m^2^ (95% CI, − 5.08 to − 0.62), and for HADS-D = 8, − 1.46 kg/m^2^ (95% CI, − 2.23 to − 0.69), and − 7.52 kg/m^2^ (95% CI, − 11.97 to − 3.07). The CBT intervention had no significant effect at the 1-year follow-up irrespective of depression scores. The group comparisons at all posttests were non-significant for patients with no symptoms of depression. In addition, patients with no symptoms of depression in the follow-up period were weight stable between the 1- and 4-year follow-ups after bariatric surgery, independent of presurgical treatment (Fig. 2).

## Discussion

This 4-year follow-up of an RCT assessed the effects of a 10-week CBT intervention aiming to reduce dysfunctional eating behaviors before bariatric surgery. Generally, the study did not demonstrate any clinically noteworthy long-term effects of the intervention on dysfunctional eating behaviors, affective symptoms, or body weight. One possible explanation may be the particularly strong effects of bariatric surgery itself on all outcomes [18, 37]. Our findings concur with the fact that the average long-term weight-reducing effect of even high-quality non-surgical obesity treatment including elements of behavioral therapy is nearly negligible as compared with the corresponding effect of gastric bypass (5% at 8 years vs 27% at 12 years) [38, 39].

A small immediate treatment effect of CBT on BMI disappeared at the 1- and 4-year follow-ups, respectively. However, explorative analyses showed a significant moderator role of depressive symptoms during the follow-up period, as CBT-treated patients with minor or considerable symptoms of depression had a significantly larger weight loss immediately after CBT before surgery (− 1.05 kg/m^2^ and − 1.46 kg/m^2^), and 4 years after bariatric surgery (− 2.85 kg/m^2^ and − 7.52 kg/m^2^), than the control group. The discrepancy between the significant immediate and 4-year effects of CBT and the lack of 1-year effect might have several explanations. First, although the CBT before surgery did not focus on weight loss, it is plausible that the considerable CBT-related improvement in eating behaviors and reduction of affective symptoms might have contributed to the small weight loss before surgery [11, 16, 17, 21]. Second, the large effect of bariatric surgery at 1 year on both groups might have overshadowed a smaller effect of CBT. Third, the declining effect of bariatric surgery and weight regain between 1 and 4 years after surgery were seen particularly in patients in the control group with symptoms of depression, while those with symptoms of depression who had received CBT seemed to have been better protected against weight regain.

Although the findings need confirmation, patients with depression are at greater risk for long-term weight regain after bariatric surgery, and presurgical CBT intervention may mitigate this problem. Further, it is plausible that a CBT intervention for patients with depression at 1–2 years following surgery might be even more efficient, as the most profound biological impacts of bariatric surgery are decreasing and emotional elements may play a bigger role in overall well-being and weight loss (Fig. 2).

The notion of a preventive effect of CBT in patients with symptoms of depression may be at odds with the fact that at the 4-year follow-up, the CBT group scored higher on the HADS-D measure than the control group. This may represent a floor effect generated by a markedly higher variance in the CBT (SD = 3.5) compared to the control group (SD = 1.9) given the generally low depression scores in both groups. Thus, scores in the CBT arm could only vary upwards. Furthermore, at the 4-year follow-up, the correlation between the residualized change score for BMI (regressing the last BMI score on BMI baseline) and HADS scores was significantly positive in the control group (*r* = .53, *P* = .002), but not in the CBT group (*r* = .18, *P* = .36).

One previous RCT reported no significant effects of adjuvant pre- and postoperative CBT on weight loss, eating habits, or physical exercise 2 years after gastric bypass [14], while another RCT demonstrated that a comprehensive lifestyle intervention program with a cognitive behavioral component before surgery did not improve weight loss 2 years after surgery [13]. However, different study designs make it difficult to compare the previous studies with the present. First, while the previous studies used CBT as a part of a lifestyle intervention program aiming to improve weight loss, the CBT intervention in the present study focused on strategies to improve dysfunctional eating behaviors. Second, the previous studies also included postsurgical treatment [13, 14].

To the best of our knowledge, this is the first long-term (> 2 years) RCT aiming to address the adjuvant somatic and psychological effects of CBT.

This study has limitations. First, as common in obesity research, the attrition rate was relatively high at 4 years, which reduced the statistical power to detect hypothesized differences in the longitudinal outcomes. The sample size was based on < 20% attrition rate at T1, it was < 4%, but attrition increased to 34% during the 4-year follow-up period. To reduce bias and loss of power, linear mixed regression models were fitted [40]. Second, all patients were recruited from a Norwegian public tertiary care center, with no personal costs for patients, which might not apply for privately financed health care systems. Third, resilience and health-related quality of life were measured only at 1 and 4 years after surgery. Fourth, all patient were white, reducing the generalizability of results to non-white populations.

Preoperative CBT was not associated with improved eating behaviors, affective symptoms, or weight loss 4 years after bariatric surgery. However, in patients with minor or considerable symptoms of depression, CBT was associated with lower body weight before and 4 years after surgery. To enhance possible direct immediate and longer term additional effects of CBT on weight loss, patients with symptoms of presurgical depression may be offered CBT targeting such symptoms as well as aspects of dysfunctional eating patterns. Additional studies are required to assess whether patients should be offered CBT before and/or after bariatric surgery, as well as which clinical aspects (e.g., eating behaviors, affective symptoms, or weight loss) the CBT should in particular address.
